# Upcycling Waste Lard Oil into Vertical Graphene Sheets by Inductively Coupled Plasma Assisted Chemical Vapor Deposition

**DOI:** 10.3390/nano7100318

**Published:** 2017-10-12

**Authors:** Angjian Wu, Xiaodong Li, Jian Yang, Changming Du, Wangjun Shen, Jianhua Yan

**Affiliations:** 1 State Key Laboratory of Clean Energy Utilization, Zhejiang University, Hangzhou 310027, China; wuaj@zju.edu.cn (A.W.); lixd@zju.edu.cn (X.L.); yzyj@zju.edu.cn (J.Y.); 2 School of Environmental Science and Engineering, Sun Yat-sen University, Guangzhou 510275, China; duchm@mail.sysu.edu.cn; 3 China United Engineering Corporation, Hangzhou 310052, China; shenwj@chinacuc.com

**Keywords:** ICP, PECVD, waste lard oil, VG, characterization

## Abstract

Vertical graphene (VG) sheets were single-step synthesized via inductively coupled plasma (ICP)-enhanced chemical vapor deposition (PECVD) using waste lard oil as a sustainable and economical carbon source. Interweaved few-layer VG sheets, H_2_, and other hydrocarbon gases were obtained after the decomposition of waste lard oil. The influence of parameters such as temperature, gas proportion, ICP power was investigated to tune the nanostructures of obtained VG, which indicated that a proper temperature and H_2_ concentration was indispensable for the synthesis of VG sheets. Rich defects of VG were formed with a high $I_{D}/I_{G}$ ratio (1.29), consistent with the dense edges structure observed in electron microscopy. Additionally, the morphologies, crystalline degree, and wettability of nanostructure carbon induced by PECVD and ICP separately were comparatively analyzed. The present work demonstrated the potential of our PECVD recipe to synthesize VG from abundant natural waste oil, which paved the way to upgrade the low-value hydrocarbons into advanced carbon material.

## 1. Introduction

Benefiting from its fascinating mechanical, electrical, optical, and thermal properties, graphene with a two-dimensional honeycomb-like framework emerges as a promising alternative to initiate a new material revolution [1]. Since the first discovery of graphene by mechanical exfoliation [2,3], various methods have been developed, such as chemical vapor deposition (CVD), exfoliation of SiC, and chemical reduction of graphene oxide (GO), aiming at various potential industrial and academic applications in energy storage and conversion, gas sensors, adsorbents, and field-effect transistors (FETs) [4,5,6]. For example, Wang et al. investigated graphene chemical vapor deposition growth on various transition metal surfaces, and found different dissociation rates of methane and diffusion growth of graphene on substrate [7]. Pei et al. reviewed the state-of-the-art status of GO reduction on both techniques and mechanisms, and discussed the effects of different reduction processes on the properties of reduced GO [8]. However, these methods are restrictive with the consideration of precursor specification, high processing temperature, heavy energy consumption, and the use of hazardous chemicals [9]. Non-sustainable and explosive petroleum gases like CH_4_ and C_2_H_2_ are usually designated as carbon precursors, which renders a higher cost of production and safety management. Hence, substantial efforts have been made to develop green and cost-efficient technologies to produce graphene.

Recently, there has been a significant increase in graphene synthesis using non-thermal plasma technologies [10]. Owing to the generation of energetic electrons and versatile active species (radicals, ions, photons, excited molecules, and atoms), non-thermal plasma affords a low temperature, high chemical reactivity, and flexible parameter tuning environment for the growth of versatile carbon materials [6,11]. As such, numerous studies have been performed to investigate the utilization of non-thermal plasma towards tailoring graphene properties for various applications. Among the various non-thermal plasma sources (e.g., gliding arc plasma, glow plasma, microwave plasma, etc.), inductively coupled plasma (ICP) is proposed to realize a controllable vertical graphene (VG) synthesis in the present work. In this stable and non-thermal equilibrium plasma environment, the growth of well-oriented vertical graphene sheets with reactive edge structure and abundant open internal channels is anticipated [10,12]. Compared with the conventional horizontal random stacking graphene sheets associated with van der Waals interactions, self-supported VG sheets are favored in practical applications because of their unique mechanical, chemical, and electrochemical properties. For example, the non-agglomerated morphology of VG gives a high specific surface area and abundant open channels between the sheets. Additionally, long, exposed, ultra-thin graphene edges on VG sheets exhibit chemical activity, making VG attractive for emerging energy and environmental application [6].

Herein, waste lard oil originated from daily cooking and oil manufacturers is introduced as a cheap and green carbon donor to synthesize VG, aiming at the revalorization of waste into high-value-added materials. Lard oil is one of most significant oils consumed in China. According to the statistics in 2002, the consumption proportion of lard oil occupied nearly 29% amongst various edible oils in daily life [13]. Hence, waste lard oil with its nature of abundance, nontoxicity, and sustainability exhibits tremendous potential as a carbon source for VG growth. In fact, natural waste has already emerged as a carbon precursor and has been investigated by researchers worldwide. Wang et al. used microwave plasma to convert waste rice husk into graphene and carbon nanotubes hybrids, and this can even be spread to other waste biomass [14]. Seo et al. used natural honey as a green carbon source to obtain VG sheets using inductively coupled plasma (ICP), and attempted application in gas- and bio-sensing [4]. Wu et al. converted waste rapeseed oil into hydrogen and carbon materials using gliding arc plasma, and analyzed the discharge characteristics in plasma [15,16].

In this paper, we firstly propose the use of waste lard oil as carbon donor for a one-step synthesis of VG sheets using inductively coupled plasma (ICP)-enhanced chemical vapor deposition (PECVD). The morphology, graphitized degree, and wettability of the obtained carbon was investigated. Furthermore, the effects of temperature, gas proportion, and ICP power on the properties of waste lard oil-based graphene were studied. Finally, a probable growth mechanism of VG is suggested.

## 2. Materials and Methods

**Figure 1:**
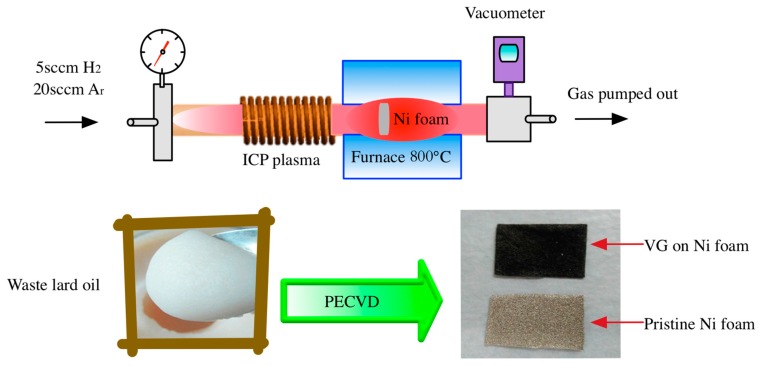
Schematic diagram of inductively coupled plasma-enhanced chemical vapor deposition (ICP-PECVD) applied for the synthesis of waste lard oil-based vertical graphene.

The growth of waste lard oil-based vertical graphene was carried out in a radio frequency (RF) PECVD system, as illustrated in Figure 1. The ICP reactor was in a cylindrical configuration, constituted by a quartz tube and 13-turns water-cooled copper antenna. Driven by a 13.56 MHz RF power source (HERO-500W, Zhongshan k-mate Electronics Co., Ltd., Zhongshan, China), plasma was generated by a time-varying electromagnetic field. A rotary pump (RVP-2, KYKY Technology Co., Ltd., Beijing, China) was used to maintain a low pressure in a range from 100 mTorr to 10 Torr. Nickel foam with purity of 99.99% was selected as a substrate for VG growth. For each test, approximately 1 mg of waste lard oil derived from a restaurant was uniformly smeared on the nickel foam. Mixture of Ar and H_2_ was purged as carrier gas to maintain a reducing atmosphere. By optimizing the growth time of VG sheets, 15 min was chosen as a suitable reaction time. As shown in Figure 1, the previous silvery white Nickel foam covering a thin oil was completely converted into a black foam with VG sheets deposited.

The surface structure and morphology of prepared VG sheets were evaluated by a field emission scanning electron microscope (FESEM, S-3700N, Hitachi, Hitachi, Japan). Raman spectroscopy (Labor Raman series, HR-800, Jobin Yvon, Paris, France) was used to characterize the graphitic character of VG sheets. Additionally, the contact angles of water droplets on the surface was measured by a digit goniometer (DropMete, A-200, MAIST Vision Inspection & Measurement Co., Ltd., Ningbo, China) to evaluate the wettability. The exhaust gas was sampled and estimated using a gas chromatograph (GC9790A, Fuli Analytical instrument, Taizhou, China) equipped with a thermal conductivity detector (TCD) for H_2_ and CO, and a flame ionization detector (FID) for hydrocarbons [11].

## 3. Results and Discussion

**Figure 2:**
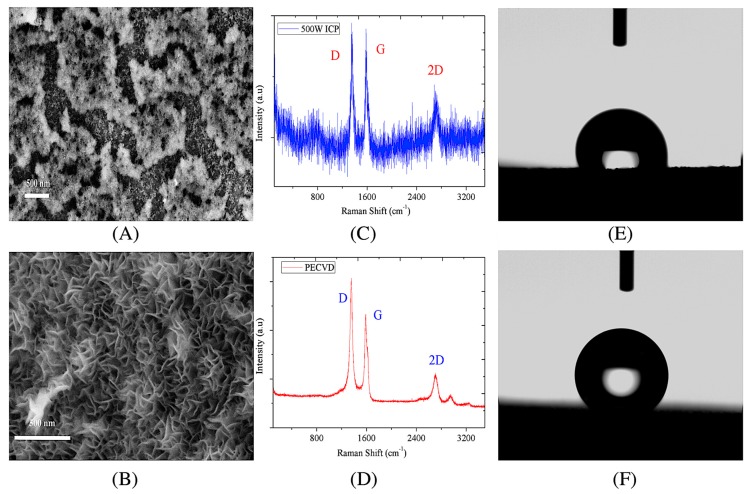
(**A**) High-resolution images; (**C**) Raman spectroscopy; (**E**) Contact angle of obtained carbon generated by ICP plasma; (**B**) High-resolution images; (**D**) Raman spectroscopy; (**F**) Contact angle of vertical graphene (VG) generated by PECVD. Power = 500 W, H_2_ = 5 sccm, Ar = 20 sccm.

In the SEM images of Figure 2A, moss-like carbon aggregate was obtained by ICP plasma, which was arranged in an inhomogeneous size distribution. Additionally, small spherical carbon nanoballs were also observed below these coagulations of carbon clusters. Without external heating, a complex and irregular morphology of solid carbon was obtained only using 500 W ICP. Through characterization by Raman spectroscopy, defects and crystallization degree of these obtained carbon could be further revealed. D peak (corresponding to the defects or amorphous carbon) at ~1350 cm^−1^, G peak (corresponding to the presence of graphitized carbon and double degenerate deformation vibrations of sp^2^ chain) at ~1580 cm^−1^, and 2D peak (corresponding to overtone of D peak) at ~2700 cm^−1^ were observed in the spectrum [17,18]. The intensity ratio of D-to-G peak ($I_{D}/I_{G}$) was used to quantify the structural purity or defect’s quantity in graphitic material, while the 2D-to-G peak ($I_{2D}/I_{G}$) indicated the layers of graphene. In Figure 2C, $I_{D}/I_{G}$ and $I_{2D}/I_{G}$ were approximately 1 and 0.92, respectively, while the intense background signal corresponded to the effect of fatty acids. It was conjectured that although ICP plasma could partly break down long aliphatic chains of oil into light molecules such as aromatic hydrocarbons, alkanes, olefins etc., it was still too low to support the self-assembly of carbon atoms [16].

In Figure 2B, well-oriented VG was formed after 15 min treatment via PECVD. Dense VG sheets with sharp edges were discernible, while the thickness and lateral size were of about 10 nm and 200 nm, respectively. Such graphene edges grew upward on the nickel foam and interweaved mutually, resulting into a porous structure and micro channels between sheets. The distribution of pore size formed by VG sheets’ edges indicates a mean diameter of 12 nm. The observed bright films covering the VG sheets was carbonic material, which was attributed to the joint effect of ICP plasma and graphene edges [19]. Accordingly, sharp and intense D peak with a high $I_{D}/I_{G}$ ratio of 1.29 is illustrated in Figure 2D, in agreement with the rich dense edges observed in SEM. The $I_{2D}/I_{G}$ of 0.45 implied few-layer sheets formed in VG [4]. In addition, D + G peak (~2940 cm^−1^, corresponding to a combination of scattering peak) and 2D’ (~3200 cm^−1^) was also observed in the second-order Raman spectrum [18].

The contact angles of water droplets on carbon obtained by ICP plasma and PECVD are comparatively analyzed in Figure 2E,F. The contact angle of water droplet on VG sheets was 141.5${}^{{}^{\circ}}$, exhibiting a better hydrophobic property than the counterpart obtained by separate ICP treatment (contact angle = 107.2${}^{{}^{\circ}}$). Such super hydrophobic property of waste lard oil-based VG could be attributed to several aspects. Firstly, a high ratio of hybridized sp^2^ bonding and π−π electrons coupling effect promoted the rejection of polar molecules like water droplets. Then, water-repellent effect was also enhanced by amounts of cavities formed by interweaved VG sheets. With the consideration of the relatively high composition ratio of N in lard oil, the heterogeneous atoms doped on the edges of VGs might also cause the hydrophobic surface performance [20]. Hence, apart from the existing reactive electro-chemical environment induced by ICP plasma, high-quality carbon (VG sheets) was also deeply dependent on external heating.

**Figure 3:**
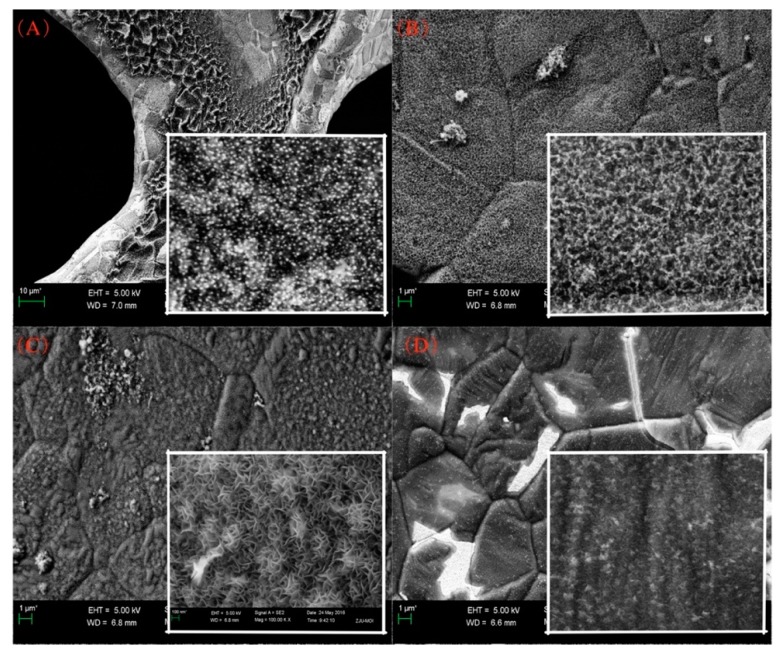
SEM images of VG sheets generated by PECVD at temperature of (**A**) 600 ${}^{{}^{\circ}}C$; (**B**) 700 ${}^{{}^{\circ}}C$; (**C**) 800 ${}^{{}^{\circ}}C$; and (**D**) 900 ${}^{{}^{\circ}}C$. Power = 500 W, H_2_ = 5 sccm, Ar = 20 sccm.

In fact, heating temperature was a significant parameter to tune the morphology of VG sheets. When temperature was at 600$\;{}^{{}^{\circ}}C$, only chopped carbon sheets were formed, and were covered by homogeneous granular particles. When the temperature was increased to 700$\;{}^{{}^{\circ}}C$, spherical carbon particles with relatively regular nanostructure were observed, which were mutually cross-linked and resulted in a dense three-dimensional network distribution. However, still no VG sheet morphology was formed. When the temperature was set at 800$\;{}^{{}^{\circ}}C$, maze-like VG sheets were generated with the lateral edge size of about 200 nm. Similar morphology of VG sheets was also obtained with pure CH_4_ as carbon precursor by ICP-PECVD [21]. Further increase of heating temperature incurred a shrinkage of lateral size and promoted the formation of larger lateral size carbon sheets. As shown in Figure 3D, poor-quality graphene sheets with extremely dense and tiny edges were formed and arranged in random orientations when the heating temperature was 900$\;{}^{{}^{\circ}}C$. Hence, the influence of temperature was prominent, which might be attributed to the surface reaction kinetics of graphene growth. Wang et al. [22] concluded that VG could not be obtained when the substrate temperature was less than 600$\;{}^{{}^{\circ}}C$. With the increase of temperature, more species for nucleation as well as nucleation sites could be generated, whereas overheated temperature led to a high degree of corrugation and density of nucleation sites, and restricted the lateral growth of VG sheets. Herein, the optimal heating temperature was 800$\;{}^{{}^{\circ}}C$ for the growth of high-quality VG sheets, with consideration of its potential application in supercapacitors, field emission, and catalysis.

**Figure 4:**
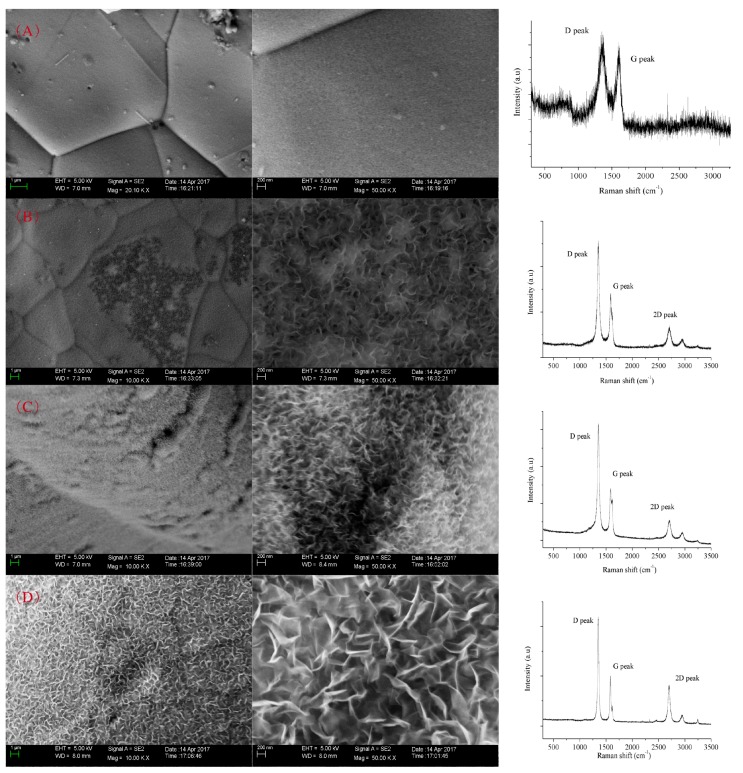
SEM images and Raman spectroscopy of VG sheets generated by PECVD with H_2_/Ar of (**A**) 0:25; (**B**) 5:20; (**C**) 10:15; and (**D**) 15:10. Power = 500 W, heating temperature = 800 °C.

The concentration of H_2_ in the carrier gas also had a crucial influence on the morphology and graphitized degree of prepared VG sheets. Without H_2_ introduced, a layer of carbon sponge was formed covering the nickel substrate instead of the generation of vertically oriented carbon sheets. Accordingly, low peak intensity and overlapping of D and G peaks was observed, with an $I_{D}/I_{G}$ ratio of about 1.11 in Figure 4A. With the increase of H_2_ concentration in carrier gas, a distinguished structure of VG sheets was achieved, which was mutually interweaved in Figure 4B,C. The lateral sizes of these edges ranged from about 100 nm to 400 nm. In the first-order Raman spectrum, the ratios of $I_{D}/I_{G}$ and $I_{2D}/I_{G}$ were 1.86–2.23 and 0.38–0.47, which implied the formation of few-layer graphene sheets with abundant edge structure. With an H_2_/Ar ratio of 3/2, the lateral size of the formed VG increased greatly and led to a high flakiness ratio, facilitating its potential application in catalysis, heat transfer, and conductivity. In Figure 4D, independent and symmetrical line profiles of D and G peaks were observed with the $I_{D}/I_{G}$ ratio as high as 2.1, which indicated a high ratio of sp^2^ structure and a high graphitized degree. The intensity of the 2D peak was enhanced with the $I_{2D}/I_{G}$ ratio of 0.82, which implied the decrease of graphene layers. Additionally, the D’ peak at ~1620 cm^−1^ was observed at the shoulder of the G peak, which was also regarded as the characteristic of VG sheets [20]. In fact, a similar VG sheet Raman spectrum was also observed with cheese as carbon source using ICP, where the ratios of $I_{D}/I_{G}$ and $I_{2D}/I_{G}$ were 1.4 and 0.95, respectively [22]. Additionally, the plasma source in PECVD would also cause great influence of $I_{D}/I_{G}$ in Raman spectrum: 0.7–2.66 for DC glow PECVD, 1.35–2.43 for microwave PECVD [23,24,25].

.

In general, H_2_ was indispensable for VG synthesis, while H_2_ in the reaction atmosphere was mainly from two sources: the initial carrier gas and gas products attributed to oil decomposition. In terms of waste lard oil conversion by PECVD, H_2_, CH_4_, and C_2_H_4_ were the major gas products, while CO, CO_2_, C_2_H_6_, and C_2_H_2_ were also detected in the exhaust gas. The generation of H_2_ in the reaction probably originated from the proton extraction from aliphatic hydrocarbon, graphene formation, or directly splitting into atoms via electron impaction [26,27]. On the other hand, the initially injected H_2_ not only contributed to total H_2_ collected at the exit, but played a significant role during reactions. This is because higher H_2_ concentration promoted the lateral growth of VG and inhibited its width increase simultaneously.

**Figure 5:**
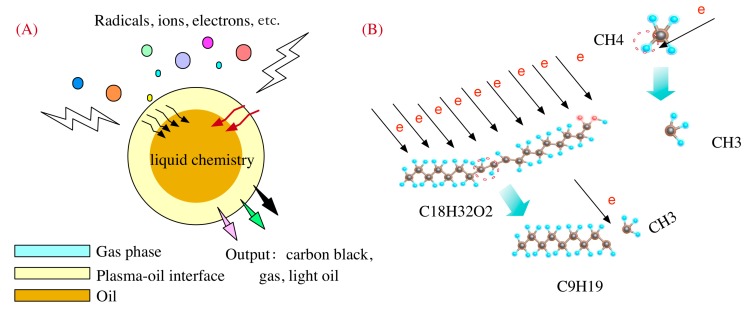
(**A**) Decomposition of waste lard oil in PECVD and the effect of electron collisions; (**B**) Breakdown of fatty acid chains induced by electron collisions.

By characterizing the VG sheets and gaseous products by PECVD, it was concluded that the conversion of waste lard oil into vertical graphene sheets and gas was a complex electro-chemical process, as shown in Figure 5. Firstly, electrons were initiated and propagated by the time-varying electromagnetic filed in ICP, which subsequently collided with *Ar* and *H*_2_ molecules to form active species such *H* radicals, *Ar* excited molecules. These active species were responsible for energy conversion as well as radical reactions [28].

(1) $$e+Ar\to Ar^{*}+e$$

(2) $$e+RCH_{2}CH_{2}CH_{3}\to RCH_{2}CH_{2}CH_{2}+H+e$$

(3) $$e+RCH_{2}CH_{2}CH_{3}\to RCH_{2}CH_{2}+CH_{3}+e$$

(4) $$Ar^{*}+RCH_{3}\to RCH_{3}{}^{*}+e$$

(5) $$Ar^{*}+RCH_{2}CH_{2}CH_{3}\to RCH_{2}CH_{2}CH_{2}+H+Ar$$

(6) $$C_{n}H_{2n-1}+H\to C_{n}H_{2n}+H_{2}$$

In addition, high temperature promoted the pyrolysis of aliphatic chains and provided a proper environment for VG growth. Compared with traditional CVD method, the temperature required for VG synthesis was greatly reduced with the help of ICP.

(7) $$C_{n}H_{2n+2}\to nC+\left(n+1\right)H_{2}$$

(8) $$C_{n}H_{2n+2}\to C_{n-m}H_{2n-2m+2}+C_{m}H_{2m}$$

On the other hand, different from conventional carbon precursors such as C_2_H_2_ or CH_4_, waste lard oil constituted by long chain fatty acids or esters was prone to be initially decomposed into various light volatiles, which resulted in a rapid increase of reaction pressure from 600 mTorr to 2 Torr [14]. Accompanied with the increase of the heating temperature to a designated temperature, carbon-containing species sourced from waste lard oil were gradually converted into amorphous carbon or nucleation sites on the catalytic nickel surface, providing a base for graphene growth. With the onset of 500 W ICP plasma, large amounts of active species such as energetic electrons, excited argon molecules, and H atoms were produced and involved in related reactions. For example, H radicals played a significant role in etching amorphous carbon and bringing away heteroatoms on VG sheets, while the collisions between other active species and light volatiles contributed to the continuous generation of graphene-building species such as C_2_ carbon radicals. In addition, under the joint effect of high temperature and reactive ICP plasma, light gaseous hydrocarbons were also detected due to the decarboxylation, decarbonylation, and C–C bond cleavage in the reaction. Additionally, the operating conditions could not only tailor the morphology of formed VG, but also influenced the distribution of gas products. For example, the proportion of CH_4_ decreased with the increase of H_2_/Ar ratio in carrier gas, whereas the proportion of C_2_ hydrocarbons such as C_2_H_4_, C_2_H_2_, and C_2_H_6_ increased in contrast.

## 4. Conclusions

In the present research, waste lard oil sourced from catering and daily home cooking exhibited its potential as a green and cheap carbon precursor to synthesize VG using ICP-PECVD. Through characterization by SEM, Raman spectroscopy, and contact angles, the optimal conditions for VG synthesis were revealed. Without the help of high temperature, moss-like carbon clusters were obtained with an $I_{D}/I_{G}$ ratio of about 1 and a contact angle of 107.2${}^{{}^{\circ}}$. It was conjectured that the energy and temperature from ICP plasma could not support the self-assembly of atoms into oriented graphene sheets. By tuning the heating temperatures, thin, few-layer, and dense-edge VG sheets were achieved at 800$\;{}^{{}^{\circ}}C$, with the ratios of $I_{D}/I_{G}$ and $I_{2D}/I_{G}=0.92$ of 1.29 and 0.45, respectively. The contact angle of water droplets on the VG surface was even more than 140${}^{{}^{\circ}}$, exhibiting a super hydrophobic property. By analyzing the exhaust gas, H_2_, CH_4_, and C_2_ hydrocarbons were detected. The joint effect of ICP and high temperature could promote the decomposition of lard oil into light molecules, and provided a suitable environment for the generation of nucleation sites, self-assembly of carbon species, as well as the subsequent growth of VG. By single-step synthesis of waste organic lard oil-based VG using ICP-PECVD, we believe that more daily waste could be exploited as sustainable carbon precursors to compensate the graphene market in the future.

## References

[1] Geim A.K., Novoselov K. (2007). The rise of graphene. Nat. Mater..

[2] Novoselov K.S., Geim A.K., Morozov S.V., Jiang F., Zhang Y., Dubonos S.V., Grigorieva I.V., Firsov A.A. (2004). Materials and methods: Electric field effect in atomically thin carbon films. Science.

[3] Seo H.K., Kim T.S., Park C., Xu W., Baek K., Baw S.H., Ahn J.H., Kim K., Choi H.C., Lee T.W. (2015). Value-added synthesis of graphene: Recycling industrial carbon waste into electrodes for high-performance electronic device. Sci. Rep..

[4] Seo D.H., Rider A.E., Kumar S., Randeniya K.L., Ostrikov K. (2013). Vertical graphene gas- and bio-sensors via catalyst-free, reactive plasma reforming of natural honey. Carbon.

[5] Pumera M. (2011). Graphene-based nanomaterials for energy storage. Energy Environ. Sci..

[6] Bo Z., Yang Y., Chen J.H., Yu K.H., Yan J.H., Cen K.F. (2013). Plasma-enhanced chemical vapor deposition synthesis of vertically oriented graphene nanosheets. Nanoscale.

[7] Wang X., Yuan Q., Li J., Ding F. (2017). The transition metal surface dependent methane decomposition in graphene chemical vapor decomposition growth. Nanoscale.

[8] Pei S., Cheng H.M. (2012). The reduction of graphene oxide. Carbon.

[9] Bazaka K., Jacob M.V., Ostrikov K. (2016). Sustainable life cycles of natural-precursor-derived nanocarbons. Chem. Rev..

[10] Prasad K., Bandara C.D., Kumar S., Singh G.P., Brockhoff B., Bazaka K., Ostrikov K. (2017). Effect of precursor on antifouling efficacy of vertically-oriented graphene nanosheets. Nanomaterials.

[11] Wu A.J., Li X.D., Yan J.H., Yang J., Du C.M., Zhu F.S., Qian J.Y. (2017). Co-generation of hydrogen and carbon aerosol from coalbed methane surrogate using rotating gliding arc plasma. Appl. Energy.

[12] Seo D.H., Han Z.J., Kumar S., Ostrikov K. (2013). Structure-controlled, vertical graphene-based, binder-free electrodes from plasma-reformed butter enhance supercapacitor performance. Adv. Energy Mater..

[13] Ma G.S., Hao L.N., Li X.P., Hu X.Q., He Y.N., Zhai F.Y., Yang X.G., Kong Y.Z. (2008). Cooking oil consumption of adults in China. Food Nutr. China.

[14] Wang Z.P., Ogata H., Morimoto S., Medina J.O., Fujishige M., Takeuchi K., Muramatsu H., Hayashi T., Terrones M., Hashimoto Y. (2015). Nanocarbons from rice husk by microwave plasma irradiation: From graphene and carbon nanotubes to graphenated carbon nanotube hybrids. Carbon.

[15] Wu A.J., Li X.D., Chen L., Du C.M., Yan J.H. (2015). Investigation of the physical properties in rotating gliding arc discharge with rapeseed oil. IEEE Trans. Plasma Sci..

[16] Wu A., Li X., Chen L., Zhu F., Zhang H., Du C., Yan J. (2015). Utilization of waste rapeseed oil by rotating gliding arc plasma. Int. J. Hydrogen Energy.

[17] Tsaneva V.N., Kwapinski W., Teng X., Glowacki B.A. (2014). Assessment of structural evaluation of carbons from microwave plasma natural gas reforming and biomass pyrolysis using Raman spectroscopy. Carbon.

[18] Jacob M.V., Rawat R.S., Bo Q.Y., Bazaka K., Kumar D.S., Taguchi D., Iwamoto M., Neupane R., Varghese O.K. (2015). Catalyst-free plasma enhanced growth of graphene from sustainbale sources. Nano Lett..

[19] Qu B., Lian X.B., Wu Q.H. (2016). Growth of three-dimensional graphene films on the Ni foil. Surf. Eng..

[20] Watanabe H., Kondo H., Sekine M., Hiramatsu M., Hori M. (2012). Control of super hydrophobic and super hydrophilic surfaces of carbon nanowalls using atmospheric pressure plasma treatments. Jpn. J. Appl. Phys..

[21] Seo D.H., Kumar S., Ostrikov K. (2011). Control of morphology and electrical properties of self-organized graphenes in a plasma. Carbon.

[22] Wang J.J., Zhu M.J., Outlaw R.A., Zhao X., Manos D.M., Holloway B.X. (2004). Synthesis of carbon nanosheets by inductively coupled radio-frequency plasma enhanced chemical vapor deposition. Carbon.

[23] Seo D.H., Yick S., Pineda S., Su D., Wang G., Han Z.J., Ostrikov K. (2015). Single-step, plasma-enabled reforming of natural precursors into vertical graphene electrodes with high areal capacitance. ACS. Sustain. Chem. Eng..

[24] Kurita S., Yoshimura A., Kawamoto H., Uchida T., Kojima K., Tachibana M. (2005). Raman spectra of carbon nanowalls grown by plasma-enhanced chemical vapor deposition. J. Appl. Phys..

[25] Ni Z.H., Fan H.M., Feng Y.P., Shen Z.X., Yang B.J., Wu Y.H. (2006). Raman spectroscopic investigation of carbon nanowalls. J. Chem. Phys..

[26] Cheng L., Qu L., Deng J.H. (2016). High-efficiency field emission from pressed nickel foam-flat graphene-vertical graphene hybrids. Mater. Lett..

[27] Idem R.O., Katikaneni S.P.R., Bakhshi N.N. (1996). Thermal cracking of canola oil: Reaction products in the presence and absence of steam. Energy Fuel.

[28] Maher K., Bressler D. (2007). Pyrolysis of triglyceride materials for the production of renewable fuels and chemicals. Bioresour. Technol..

